# Is Simple Reimplantation a Viable Option in Pediculated Auricular Avulsions? A Systematic Review of the Literature

**DOI:** 10.3390/cmtr18030036

**Published:** 2025-08-27

**Authors:** Jose Carlos Román Padilla, Luis Ortiz Peces, Pol Alavedra Martínez, Jose Luis Cebrián Carretero

**Affiliations:** Department of Oral and Maxillofacial Surgery, La Paz University Hospital, 28046 Madrid, Spain; luisort98@gmail.com (L.O.P.); alavedramartinezpol@gmail.com (P.A.M.); josel.cebrian@salud.madrid.org (J.L.C.C.)

**Keywords:** auricle, auricle surgery, ear injuries, replantation, wound healing

## Abstract

Auricular avulsion injuries are rare, and microvascular reimplantation is considered the preferred treatment according to current literature. However, when a small skin pedicle is preserved, non-microvascular reattachment techniques may offer comparable outcomes. This systematic review aims to assess whether these techniques could represent a viable alternative. We analyzed 32 cases of pedicled auricular avulsion reported in 16 articles, focusing on patient demographics, injury mechanisms, pedicle characteristics, venous congestion, and postoperative management. Venous congestion occurred in 11 patients, with a significantly higher risk in narrower pedicles (mean width 9.82 mm; 95% CI: 4.75–14.89; *p* = 0.025). Prophylactic heparin significantly reduced this risk (*p* = 0.007). Other interventions—leech therapy and hyperbaric oxygen—lacked sufficient data for firm conclusions. Most cases achieved graft survival; necrosis occurred in some, and only two patients required additional surgery. Non-microvascular techniques appear to be a viable alternative to microvascular reimplantation, with similar results and potentially fewer complications. Venous congestion remains the main challenge, requiring active management and hospitalization for monitoring. Limited case series and publication bias still hinder the development of standardized guidelines.

## 1. Introduction

Auricular avulsion lesions are relatively uncommon in emergency departments within our environment. To date, the literature on this subject remains limited, making it difficult to establish a definitive prognosis or standardized management protocol for these types of injuries. There have been few attempts to gather data on cases of complete auricular avulsions, with notable contributions from Armin Steffen et al. in 2006 and Andrew D. Gailey et al. in 2020 [1,2]. Both authors presented systematic reviews summarizing the reimplantation techniques used for complete auricular avulsions. Collectively, their findings indicate that the microvascular approach for the reimplantation of complete avulsions is the most effective treatment, providing superior outcomes in terms of both cosmetic restoration and graft survival.

In his study, Andrew D. Gailey further reported that aesthetic outcomes following reimplantation were significantly enhanced when a cutaneous pedicle was preserved between the avulsed fragment and the pinna insertion site [2]. This observation highlights the need to adapt treatment strategies due to the unique prognostic implications of this subgroup of lesions. A review of the current literature reveals that some authors advocate for microvascular reimplantation even in cases where a cutaneous pedicle is present. However, this technique is not without its challenges. It is widely acknowledged that microsurgical approaches are not always feasible and are associated with extended operative times—typically averaging around six hours—and increased hospital stays, averaging over 11.4 days. Furthermore, these interventions commonly involve the need for several blood transfusions [3,4]. Additionally, the incidence of venous congestion remains a common concern, with some authors considering it a normal postoperative occurrence rather than a complication.

Alternatively, several authors have explored the use of a simpler, non-microvascular reimplantation technique for patients with pedicled auricular avulsions [5,6,7,8,9,10,11,12,13,14,15,16,17,18,19,20]. Their findings suggest that, in most cases, this approach yields outcomes comparable to those achieved through microvascular techniques. This has led some to question whether non-microvascular reimplantation might serve as a viable alternative, particularly when a pedicle is present, given the potential complications associated with the more complex microsurgical technique.

The focus of this review, unlike others previously described in the literature [12,19,20], is to place particular emphasis on postoperative care and the anti-congestive measures necessary for flap survival. To the best of our knowledge, no systematic review has been conducted on this subject to date. The aim of this review is to illuminate the postoperative management of pedicled auricular avulsions and, more importantly, to assess whether the simpler reimplantation technique could serve as a reliable treatment option for these patients.

## 2. Materials and Methods

A systematic search was conducted using PubMed and the library database of our institution to identify cases of auricular avulsion with a preserved skin pedicle treated by direct reattachment (simple suture) without microsurgical techniques, regardless of clinical presentation (ischemia, congestion, capillary refill, or bleeding). The search strategy employed the following terms: (ear [mesh] OR auricle [mesh] OR pinna [MeSH Terms]) AND (Amputation, Traumatic/surgery [mesh] OR reattach* [tiab] OR avul* [tiab] OR trauma [mesh]) AND (partial [Text Word] OR incomplete [Text Word] OR subtotally [Text Word] OR near [Text Word]) AND English [lang] AND humans [mesh]. This systematic review was conducted in accordance with the PRISMA 2020 guidelines. This review was not registered in any protocol registry.

**Figure 1:**
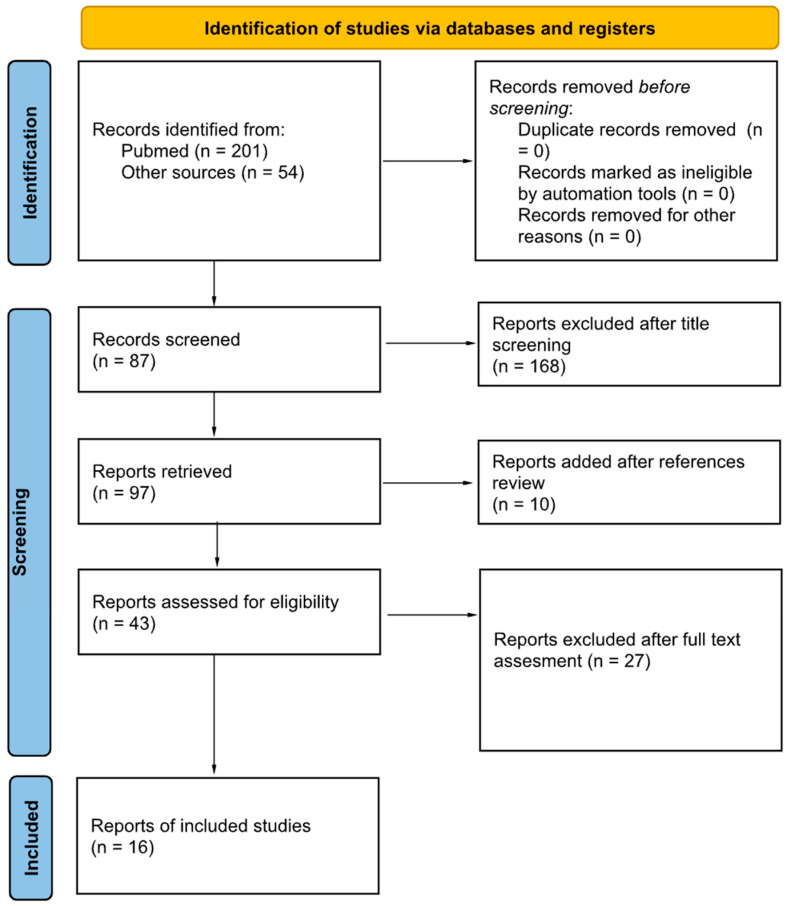
PRISMA diagram with search criteria and exclusion process.

This search yielded 201 results from PubMed and 54 results from our center’s library [Figure 1]. Studies were included if they involved pedicled auricular avulsions treated with non-microvascular reimplantation in humans and published in English. Exclusion criteria included non-acute cases, non-human studies, or reviews. A preliminary screening of titles was conducted, excluding those articles that focused on unrelated topics, did not address auricular avulsion lesions, or were not concerned with the acute management of pinna avulsion injuries. Following this initial screening, 87 articles were retrieved, and their abstracts were reviewed. Additionally, 10 more articles were identified through an exhaustive review of the references in the aforementioned studies. After evaluating the abstracts, 43 articles remained for full-text review, of which 16 were included in the quantitative analysis of our systematic review (Figure 1).

All studies responding to the inclusion criteria underwent data extraction from the patients of the following parameters: age (years), sex, injury etiology, location of the base of the pedicle, total width of the pedicle (mm), the development of venous congestion, the post-operative treatment employed, and whether this treatment was initiated before or after developing the congestion. Venous congestion was diagnosed based mainly on three clinical signs consistently reported across the included studies: progressive dark discoloration, swelling, and venous oozing, typically within the first 24–72 h postoperatively. However, variations in the exact diagnostic thresholds and lack of a description of clinical symptoms across most reports present a limitation of this review and may influence the characterization of the 11 congested auricles described. Statistical analysis was performed to see if the pedicle width is associated with the occurrence of graft congestion; for this purpose the Mann–Whitney U was used as a non-parametric alternative due to the small sample size and non-normal distribution of pedicle width data. Cases with missing data concerning pedicle width were excluded from quantitative analysis (*n* = 2). The use of prophylactic heparin was also assessed to see if it lowered the risk of venous congestion (Fisher’s exact test). Results were statistically significant for *p* values below 0.05. Cases with missing demographic or etiological data (marked as ‘NR’ in Table 1) were excluded from subgroup analyses but included in the overall descriptive statistics when pedicle size and outcomes were available. No imputation was performed given the small sample size. Sensitivity analyses excluding all cases with missing variables were conducted, and results regarding pedicle width and venous congestion remained consistent. Due to the nature of the data (mostly case reports and small series), a formal risk of bias assessment was not feasible. This systematic review was not registered in any protocol registry.

## 3. Results

**Figure 2:**
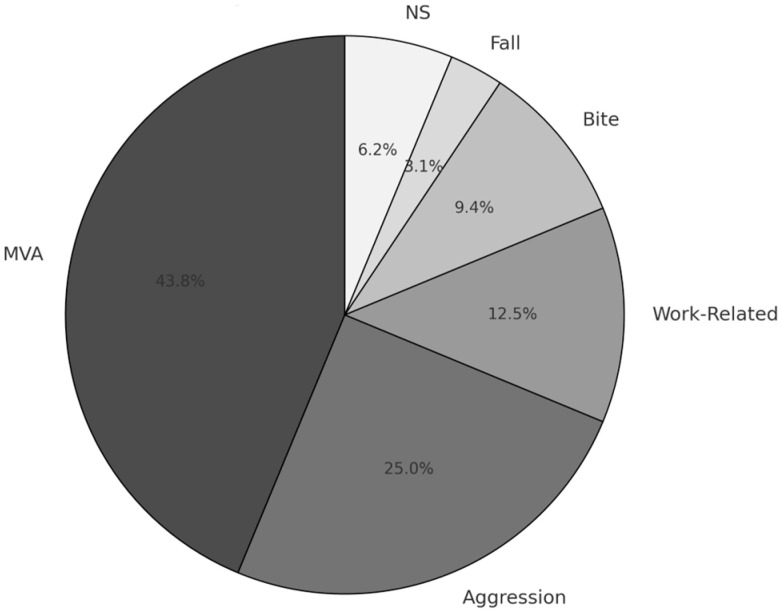
Distribution of etiology of lesion. MVA: Motor Vehicle Accident, NS: Non specified.

The results indicate that, according to the literature, 32 patients have undergone simple reimplantation procedures for cases involving pedicled avulsion. These 32 cases are reported across 16 articles published between 1968 and 2023. The average age of the patients was 31.04 years, with 14% female and 86% male representation. The most prevalent cause of injury was motor vehicle accidents (*n* = 14), followed by assault (*n* = 8) and work-related incidents (*n* = 4) (Figure 2). The mean pedicle width was 14.12 mm, with inferior pedicles (*n* = 17) being more common than superior pedicles (*n* = 12). Additionally, two cases involved both superior and inferior pedicles (*n* = 2).

**Figure 3:**
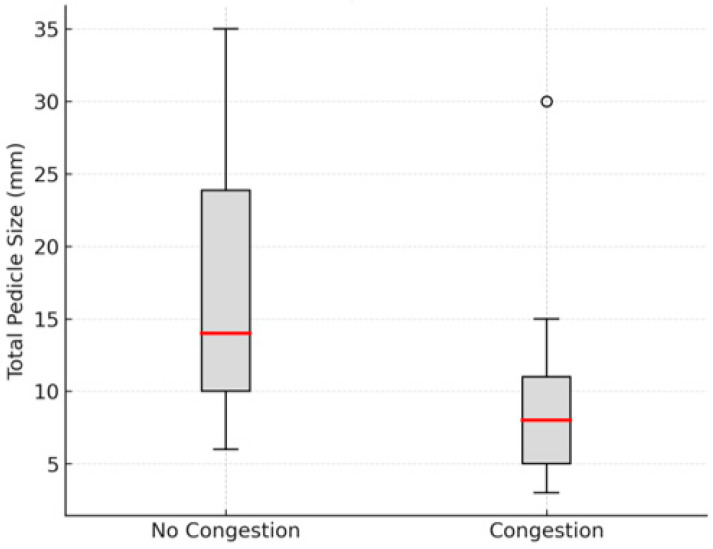
Boxplot illustrating the distribution of total pedicle size (mm) in cases with and without venous congestion. Median pedicle size was significantly smaller in cases with congestion compared to those without (Mann–Whitney U test, *p* = 0.020).

Among the 32 patients treated, 11 developed venous congestion in the reimplanted graft, although this complication resolved in all cases. The mean pedicle width was 16.61 mm (95% CI: 12.05–21.16) in cases without venous congestion and 9.82 mm (95% CI: 4.75–14.89) in cases with congestion. The difference was statistically significant (Mann–Whitney U = 157.0, *p* = 0.025) (Figure 3). This suggests that patients with auricular avulsions and a skin pedicle narrower than 14.89 mm faced a significantly higher risk of developing venous congestion.

Regarding adjuvant treatment, most authors opted for prophylactic measures prior to the onset of venous congestion signs (*n* = 13), while others initiated treatment once congestion was established, either immediately postoperatively or in the following days (*n* = 8). Additionally, some authors did not administer any treatment or did not specify the approach taken (*n* = 11). The most used regimen was prophylactic administration of heparin (*n* = 11), which appeared to be more effective in preventing venous congestion compared to other measures (*p* = 0.007). Other prophylactic measures, including leech therapy, intravenous dextran, cryotherapy, and hyperbaric oxygen therapy, were also employed [5,10,12]. However, the sample sizes for these treatments were too small to draw definitive conclusions regarding their efficacy.

A wide range of treatments have been utilized in cases where venous congestion occurred, including leech therapy (*n* = 3), low molecular weight heparin (LMWH) (*n* = 2), intravenous dextran (*n* = 1), hyperbaric oxygen therapy (*n* = 1), scalpel incisions in the congested areas (*n* = 2), pentoxifylline (*n* = 1), and vitamin E (*n* = 1) [7,10,11,13,18,19].

All the cases described resulted in a satisfactory cosmetic outcome and complete graft survival. However, some patients (*n* = 7) experienced varying degrees of necrosis, particularly in the lobule area (*n* = 3) in superiorly pedicled lesions and in the root of the helix (*n* = 2) in inferiorly pedicled lesions. Most of the included studies did not use standardized metrics to describe necrosis (e.g., affected surface area or a formal classification system). Few studies like Komorowska-Timek et al. quantified necrosis, reporting a 10 mm extension, while most of the remaining studies described only the location of the necrotic areas. Likewise, ‘satisfactory cosmetic outcome’ was defined subjectively by the original authors without standardized evaluation methods. For the purposes of this review, we considered the cosmetic outcome satisfactory when the ear preserved its overall shape and did not require major reconstructive procedures, with only two patients (6.2%) requiring secondary surgery for partial lobule necrosis.

## 4. Discussion

Auricular avulsions, while relatively uncommon, are of significant importance due to their potential impact on aesthetic outcomes. These lesions need effective management in order to achieve optimal results with a single-stage intervention.

The most widely accepted method in the literature for treating auricular avulsions is microvascular reimplantation, particularly in cases where a pedicle is available for anastomosis. This approach is favored for its aesthetic outcomes and low risk of necrosis [1,2,3,4]. However, as highlighted in this review, some authors describe successful results using non-microvascular approaches for reimplanting auricles presenting with an available skin pedicle, raising the question of whether these methods could be superior to microvascular reimplantation.

When comparing simple reimplantation to microvascular techniques in terms of cosmetic results, evidence suggests no significant difference between the two. The sole presence of the skin pedicle itself appears to improve clinical outcomes, as seen in the systematic review conducted by Gailey et al. (2020) [2]. In this review they analyzed outcomes of various surgical techniques for auricular avulsions, primarily focusing on complete avulsions but also including some pedicled cases. The authors classified aesthetic outcomes using a subjective five-point scale. Pedicled cases achieved an average score of 3.74, compared to 3.50 for microvascular cases. Although the study was not specifically designed to evaluate pedicled injuries, these findings suggest that, in terms of cosmetic outcomes, direct reattachment over a pedicle may yield results comparable to those obtained with microvascular repair. However, as discussed, the use of microvascular techniques increases operative time to approximately six hours, extends hospital stays to an average of 11.4 days, and increases the need for blood transfusions [3,4]. Furthermore, performing venous anastomosis does not appear to reduce the incidence of venous congestion [2,4,21]. Given these considerations, simple reimplantation presents as a more viable, less invasive alternative, although further observational studies are needed to draw definitive conclusions.

We have seen that there are two primary factors contributing to graft loss in auricular avulsion cases: ischemia and post-reimplantation complications, with venous congestion being the leading cause. Park et al. provided an in-depth analysis of the vasculature of the auricle, detailing the helical arcade, a vascular anastomosis between the posterior auricular artery and the superficial temporal artery that irrigates the external part of the ear [22]. This anatomical characteristic likely accounts for the ability of auricular grafts in the reviewed cases to maintain perfusion through the skin pedicle, thereby minimizing the occurrence of ischemic complications. Management should, therefore, prioritize preventing and treating venous congestion, the other key cause of graft loss in auricular avulsions.

There have been many anticongestive measures described, as seen in Table 1. The primary aim of these measures is to buy some time for the auricle to establish a new venous drainage, which typically occurs within four days for partial amputations and up to ten days for complete avulsions [23,24]. Also, proper debridement to facilitate neo-vascularization is crucial [9]. As seen in this review, systematic implementation of heparin-based anticoagulant protocols is an effective strategy in preventing venous congestion. Albdour et al. (2021) described a structured protocol of subcutaneous and intradermal low-molecular-weight heparin (LMWH), combined with superficial incisions and local wound care, tapering the dose over 10 days (4000 IU/day to 1000 IU/day), which achieved full resolution without transfusion or infection [18].

Once congestion occurs, leech therapy and making small incisions in the most congested areas have become widely accepted practices, though there is still no standardized protocol due to limited evidence [10,11,18,19,25]. In 2018 Facchin et al. highlighted medicinal leech therapy as a salvage technique when venous outflow is absent. In their article they proposed intensive application (every 4 h) during the first 4–5 days, combined with LMWH and aspirin, followed by gradual tapering until venous channels form [25]. However, although leeching is effective, it often requires blood transfusions and strict antibiotic prophylaxis [25]. Other treatments, such as hyperbaric oxygen therapy, intravenous dextran, vasodilators, cryotherapy, and vitamin E, have also been reported, but there is insufficient evidence to draw firm conclusions regarding their efficacy, and their specific administration protocols are rarely detailed in the literature. Due to the small sample sizes, no definitive conclusions can be drawn regarding the most effective method. However, based on the available literature, it appears that combining an LMWH protocol with leech therapy, along with performing incisions in the most congested areas, may represent the most promising strategy [18,25].

In our series, 11 out of 32 patients (34.4%) developed venous congestion in the reimplanted auricular graft. Furthermore, we found that smaller pedicles were significantly more likely to develop congestion when compared to larger ones (*p* = 0.02). This suggests that pedicles narrower than 14.89 mm carry a higher risk of venous congestion. For these patients, it may be advisable to intensify anticongestive measures from the start, using a combination of strategies to lower the risk of this complication.

Most authors agree that hospitalization for 4 to 6 days is necessary, as this is the estimated time required for the formation of new venous drainage and a reduced risk of venous congestion. Regarding postoperative care, edema has been reported to hinder the rapid blood flow essential for wound healing. The use of a compression splint may serve as an effective measure to optimize perfusion to the graft, as well as to prevent accumulation of blood within the perichondrium of the ear [9].

In conclusion, auricular injuries present in a variety of forms, with most being simple wounds that can be managed without specialist intervention. However, more complex cases, such as auricular avulsions, highlight the divergence in management strategies. This variation is primarily attributed to the limited number of published cases and the potential publication bias. The majority of reports on auricular avulsions consist of case reports or small case series, making it uncommon to find publications where reimplanted grafts do not survive. This limitation poses challenges in the development of standardized clinical protocols for managing auricular avulsions and complicates the accurate assessment of success rates for the interventions used.

## 5. Conclusions

Auricular avulsion injuries, though relatively rare, present significant challenges due to their potential impact on both functional and aesthetic outcomes. In this review we highlight that non-microvascular techniques yield favorable results in the presence of a skin pedicle, suggesting that less invasive approaches could be considered as viable alternatives to a microvascular approach. Postoperative care is critical, with venous congestion being the leading complication. Preventive measures such as heparin-based anticoagulant protocols have shown effectiveness, while treatments for established venous congestion, including leech therapy and small incisions in congested areas, are commonly employed. Given the lack of standardized protocols and the limited scale of current evidence, these conclusions should be interpreted with caution, and prospective multicenter studies are warranted to validate the safety and effectiveness of non-microvascular reimplantation in this setting. Ultimately, we emphasize the importance of optimizing both surgical and postoperative strategies for the best possible outcomes in these complex cases.

## Figures and Tables

**Table 1:** Reported cases of pedicled pinna avulsions treated with non-microsurgical replantation. HBOT: hyperbaric oxygen therapy, Inf: inferior based, LMWH: low molecular weight heparin, MVA: motor vehicle accident, NR: non-reported, Sup: superior based, UFH: unfractioned heparin, WR: work-related.

Author and Year	Age (Years)	Sex	Cause	Location and Total Pedicle Size (mm)	Pedicle Congestion	Medical Treatment Timing (Pre/Post Congestion)	Adjuvant Therapy
**L. Clodius et al. 1968 [5]**	NR	M	MVA	Sup, 30 mm	No	Pre	LMWH, Cryotherapy
	NR	M	MVA	Inf, 35 mm	No	Pre	LMWH, Cryotherapy
**Tomono T and Hirase, 1980 [6]**	6	M	NR	Inf, 30 mm	No	Pre	LMWH, Niacin
	6	M	NR	Inf, 10 mm	No	Pre	LMWH, Niacin
**Berstein et al., 1982 [7]**	28	F	Bite	Sup, 10 mm	Yes	Post	UFH, Cryotherapy, Dextran, Incisions
**Tunc Safak et al., 1998 [8]**	40	M	MVA	Sup, 3 mm	Yes	None	None
**Takatoshi Yotsuyanagi et al., 2001 [9]**	42	F	WR	Sup, 10 mm	No	None	None
**Komorowska-Timek et al., 2005 [10]**	35	M	WR	Sup and Inf, 7 mm	Yes	Pre	Leech, Dextran, HBOT
**Detlev Erdmann et al., 2008 [11]**	23	F	MVA	Sup, 15 mm	Yes	Post	Leech
	3	M	Fall	Sup, 8 mm	Yes	Post	Leech
	52	M	WR	Sup, 5 mm	Yes	Post	Leech
**Ozcelik D et al., 2009 [12]**	36	M	MVA	Sup, 6 mm	No	Pre	Dextran
**Bada and Pope, 2013 [13]**	4	M	Bite	Inf, 30 mm	Yes	Post	HBOT
**Aremu SK et al., 2014 [14]**	12	M	MVA	Sup, 20 mm	No	None	None
	31	M	Bite	Inf, - mm	No	None	None
	45	M	Assault	Sup, - mm	No	None	None
**Kemaloglu CA et al., 2015 [15]**	57	M	WR	Inf, 5 mm	Yes	Pre	LMWH
**Zhang C, et al., 2018 [16]**	16	M	Assault	2 Inf, 8 mm	No	Pre	LMWH
**Mauro D’Arcangelo et al., 2020 [17]**	34	M	MVA	Sup, 7 mm	No	Pre	LMWH
	31	M	MVA	Inf, 25 mm	No	Pre	LMWH
	50	M	Assault	Sup and Inf, 20.5 mm	No	Pre	LMWH
	34	M	Assault	Inf, 10 mm	No	Pre	LMWH
	32	M	MVA	Inf, 6 mm	No	Pre	LMWH
**Mahammad Albdour et al., 2021 [18]**	55	M	MVA	Sup, 5 mm	Yes	Post	LMWH, Incisions
**Karam A. Allam et al., 2021 [19]**	19	M	MVA	Inf, 8 mm	Yes	Post	Incisions
	25	M	Assault	Inf, 9 mm	No	None	None
	45	M	MVA	Inf, 12 mm	Yes	Post	Pentoxifilin, Vit E
	33	M	Assault	Inf, 14 mm	No	None	None
	27	F	Assault	Inf, 10 mm	No	None	None
	20	M	MVA	Inf, 16 mm	No	None	None
	12	M	MVA	Inf, 19 mm	No	None	None
**W. Dini Widiarni et al., 2023 [20]**	28	M	Assault	Inf, 30 mm	No	None	None
